# A brighter shade of future climate on Himalayan musk deer *Moschus leucogaster*

**DOI:** 10.1038/s41598-023-39481-z

**Published:** 2023-08-07

**Authors:** Kumar P. Mainali, Paras Bikram Singh, Michael Evans, Arjun Adhikari, Yiming Hu, Huijian Hu

**Affiliations:** 1 National Socio-Environmental Synthesis Center, Annapolis, Maryland USA; 2 Conservation Innovation Center, Chesapeake Conservancy, Annapolis, Maryland USA; 3 Guangdong Key Laboratory of Animal Conservation and Resource Utilization, Guangdong Public Laboratory of Wild Animal Conservation and Utilization, Institute of Zoology, Guangdong Academy of Sciences, Guangzhou, China; 4 Biodiversity Conservation Society Nepal, Bagdol, Lalitpur, Nepal; 5 Environmental Science and Policy Dept., George Mason University, Fairfax, VA USA; 6 Department of Natural Resource Ecology and Management, Oklahoma State University, Stillwater, OK USA

**Keywords:** Ecology, Climate sciences, Ecology

## Abstract

Himalayan musk deer (*Moschus leucogaster*) is classified as an endangered species by IUCN with a historically misunderstood distribution due to misidentification with other species of musk deer, *Moschus* spp. Taking advantage of recent genetic analyses confirming the species of various populations in Nepal and China, we produced an accurate estimate of the species’ current and future distribution under multiple climate change scenarios. We collected high-quality occurrence data using systematic surveys of various protected areas of Nepal to train species distribution models. The most influential determinants of the distribution of Himalayan musk deer were precipitation of the driest quarter, temperature seasonality, and annual mean temperature. These variables, and precipitation in particular, determine the vegetation type and structure in the Himalaya, which is strongly correlated with the distribution of Himalayan musk deer. We predicted suitable habitats between the Annapurna and Kanchenjunga region of Nepal Himalaya as well as the adjacent Himalaya in China. Under multiple climate change scenarios, the vast majority (85–89%) of current suitable sites are likely to remain suitable and many new areas of suitable habitat may emerge to the west and north of the current species range in Nepal and China. Two-thirds of current and one-third of future suitable habitats are protected by the extensive network of protected areas in Nepal. The projected large gains in suitable sites may lead to population expansion and conservation gains, only when the threat of overexploitation and population decline is under control.

## Introduction

The Himalayan musk deer (*Moschus leucogaster*) represents a rare example of a convergence of crises. The species is endangered due to intense pressure from poaching and habitat degradation^1,2^. It inhabits a region of the world that is undergoing rapid climate change^3^. As the intersection of climate change, habitat degradation and poaching is pushing the species to the verge of extinction, conservation efforts have the potential to reverse this process^4–6^. However, taxonomic misidentification among Himalayan musk deer and closely related species has historically prevented an accurate estimation of the species’ current distribution. In order to effectively conserve the species, conservationists need quality data to understand where the species exists and where it is likely to exist under future climate scenarios.

**Figure 1:**
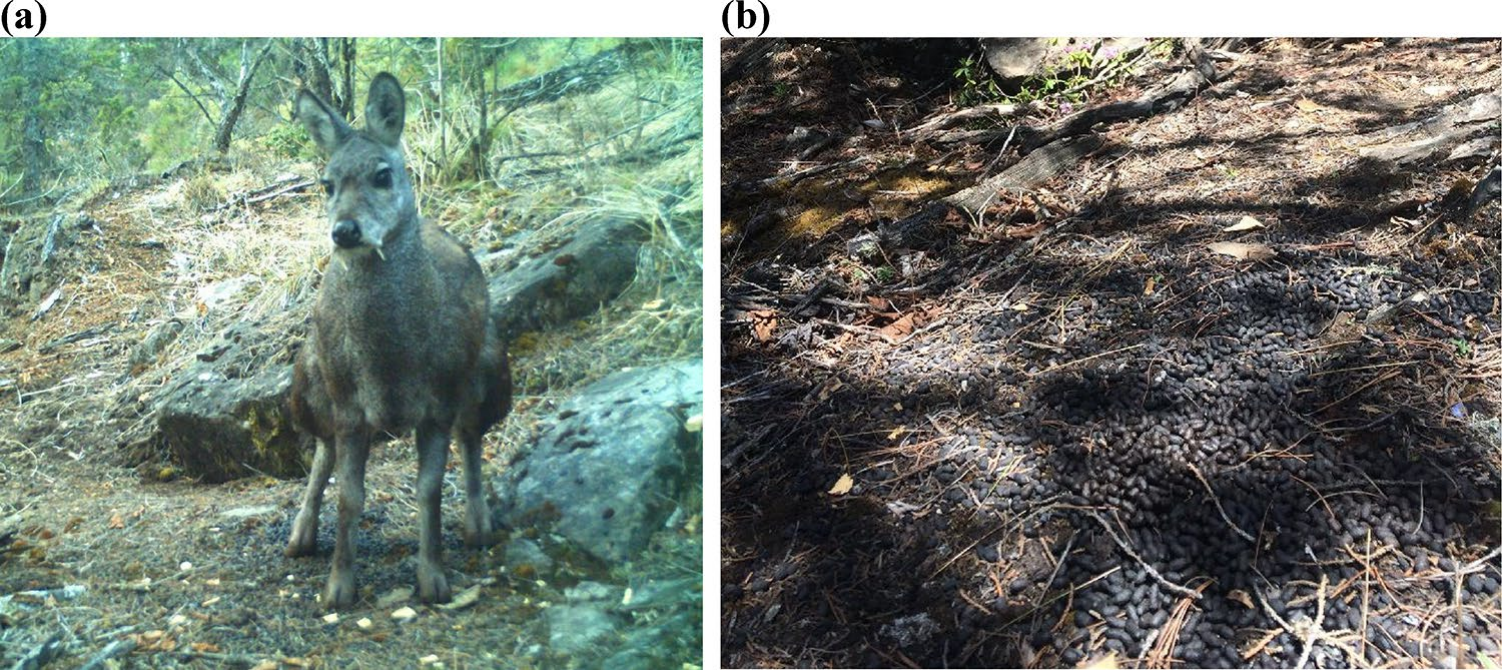
Himalayan musk deer (**a**) and its latrine site showing its characteristic pellets (**b**). Both pictures were taken in Neshyang Valley, Annapurna Conservation Area, Manang in May of 2021 by a camera trap installed by Paras Bikram Singh.

Himalayan musk deer (Fig. 1) is one of the seven species of musk deer that are endemic to the mountains of thirteen countries in Asia^2^. Himalayan musk deer, along with closely related Alpine musk deer (*Moschus chrysogaster*), Kashmir musk deer (*Moschus cupreus*) and Black musk deer (*Moschus fuscus*) inhabit the valleys and slopes of Himalaya^2,7–9^. All species of musk deer are classified as endangered by the IUCN (International Union for Conservation of Nature)^10–12^. The most significant threat to all of these species is poaching for their musk, the use of which likely began around the fifth century in Asian traditional medicines^1,13^. Accelerated poaching in the past two decades resulted in a dramatic decline in the population of Himalayan musk deer to half of the historic population, confining most of the remaining populations to protected areas^11^. A greatly reduced and fragmented range makes the species more vulnerable to the rapidly changing climate of the Himalaya which includes the mountain range spanning from China on the east to Bhutan, Nepal, India, Pakistan and Afghanistan on the west^14^. Increased precipitation, rising temperatures and melting glaciers have already been observed in the Himalaya and such climatic trends and anomalies have been predicted to be more severe in the future^15–17^.

Conservation efforts to protect Himalayan musk deer from these multidimensional threats have been inhibited by past failure to properly distinguish various species of musk deer in the Himalaya. For example, reliance on pelage color to distinguish species resulted in identification errors because of variation among individuals within a species and within individuals between seasons^9,18^. Fortunately, recent genetic studies of several populations in various parts of the Himalaya have conclusively identified different species of musk deer that were thought to be the same species^9^. For example, a pair of genetic analyses^9,19^ confirmed that all musk deer found in Manang District (to the north-east of Annapurna Himalaya), Kaski District (to the south of Annapurna Himalaya), and Gaurishankhar Conservation Area (GCA) are Himalayan musk deer. These studies also genetically confirmed that Kashmir musk deer exist to the west of Annapurna Himalaya, starting from Mustang District up to Afghanistan including Uttarakhand, India along the Himalaya^9,20,21^. The districts of Manang, Kaski, and Mustang fall in Annapurna Conservation Area (ACA). These new studies not only helped with the identification of the species, but also added new ecological insight about range boundaries.

A handful of prior studies on the distribution of musk deer could not take advantage of these recent genetic identification of the populations. For instance, Lamsal et al*.*^22^ incorrectly assumed that the musk deer populations in Annapurna Conservation Area and Gaurishankhar Conservation Area were of *Moschus chrysogaster*. Consequently, Lamsal et al.^22^ predicted that *M. chrysogaster* range encompasses the entire Nepal Himalaya. However, a handful of recent studies using mitochondrial DNA (mtDNA) have conclusively shown that musk deer populations from Himalaya (Nepal and India) are of *M. leucogaster* and *M. cupreu*s^9,19,23^. Another mtDNA analysis indicates that *M. chrysogaster* is endemic to China^24^, and so not found in Nepal. Therefore, Lamsal et al.^22^ used the occurrences of other musk deer species as *M. chrysogaster*. Another pair of recent studies by Khadka et al.^25^ and Khadka and James^26^ predicted that vast areas of the Himalaya including barren high-elevation mountains and dry Tibet currently harbor suitable sites for Himalayan musk deer. It is widely accepted that Himalayan musk deer live primarily in forested areas in the Himalaya^27–29^ and not alpine areas higher than 6000 m ASL or the deserts of Tibet. The problematic distributions predicted in these prior studies resulted at least partly from taxonomic misidentification, which until recently was somewhat unavoidable.

A standard analysis of species distribution includes associating species occurrence locations to environmental covariates, and using these relationships to predict the likelihood of occurrence in all habitats^30,31^. Such occurrence records typically come from opportunistic sightings of animals compiled by different experts and amateurs at different times. Public databases (e.g., Global Biodiversity Information Facility) hosting such records conveniently provide the aggregated occurrences to modelers. As a result, it is likely that some areas are over-represented and some under-represented. Such spatial bias has multiple detrimental effects to reliable species distribution modeling^32–34^ because an important assumption of these models is that the distributional extent of a species is either randomly or systematically sampled for occurrence records^35^. Opportunistic sightings of a species may also create sampling bias among environmental covariates, as the locations in which individuals are likely to be observed (e.g., travel corridors near human access) may not necessarily be those in which they spend the most time. On the contrary, latrines are set up in the habitat that can be easily detected by conspecifics^36^. Each individual, regardless of sex, develops a latrine in its home range (13–14 hectares)^37,38^. Males are highly territorial and their home ranges do not overlap^37^. Because latrines are set up within a home range at a location that is highly likely to be detected by other animals, the latrines are consistently found in the suitable habitats of musk deer^27,28,39^. Consequently, many previous researchers, such as Green^40^, Green ^41^, Shrestha and Meng^39^, Shrestha and Moe^27^, Singh et al.^42^, used latrine sites as a reliable proxy of musk deer distribution. Latrines (Fig. 1b) are preferably constructed close to trees and under canopy^27,28,40^. They can be identified easily; they have heaps of old and fresh pellets which are characterized by their musky smell, cylindrical shape and a size smaller than the pellets of goat (*Capra aegarus*), as observed by several prior researchers^27,28,42,43^.

In our study, we develop an updated estimate of the range of Himalayan musk deer using improved occurrence and range data over historical efforts. We obtained quality occurrence records of Himalayan musk deer from nine protected areas of Nepal, China and Bhutan. This occurrence data is superior to the previous studies modeling Himalayan musk deer distribution for three reasons. First, we collected the majority of occurrence data (90%) using systematic sampling minimizing the chances of sampling bias. A small fraction of occurrences were obtained from non-systematic surveys (8% with expert guided search and 2% opportunistic sightings) because that was the only way to represent three protected areas in our sampling locations for model training. Second, we obtained occurrences from only highly suitable sites because we searched for latrine locations of the animals; latrine sites are also confirmed and irrefutable evidence of species occurrence. Finally, we took advantage of the recent genetic findings about species identification of musk deer populations from various locations to survey our species of interest. This study has the following objectives: (1) model the distribution of Himalayan musk deer to predict habitat suitability under current and future climate scenarios, (2) determine how the size of suitable habitat changes over time, (3) measure the efficacy of protected areas in conservation at present and in future, and (4) identify locations where the species is likely to survive through time (climatic refugia). By accurately delineating the distribution of Himalayan musk deer, this study will assist IUCN efforts to accurately assess the status of the species. Future projections of Himalayan musk deer range can facilitate proactive conservation planning to ensure sufficient habitat is protected for Himalayan musk deer in the face of climate change.

## Results

### Important predictors

**Figure 2:**
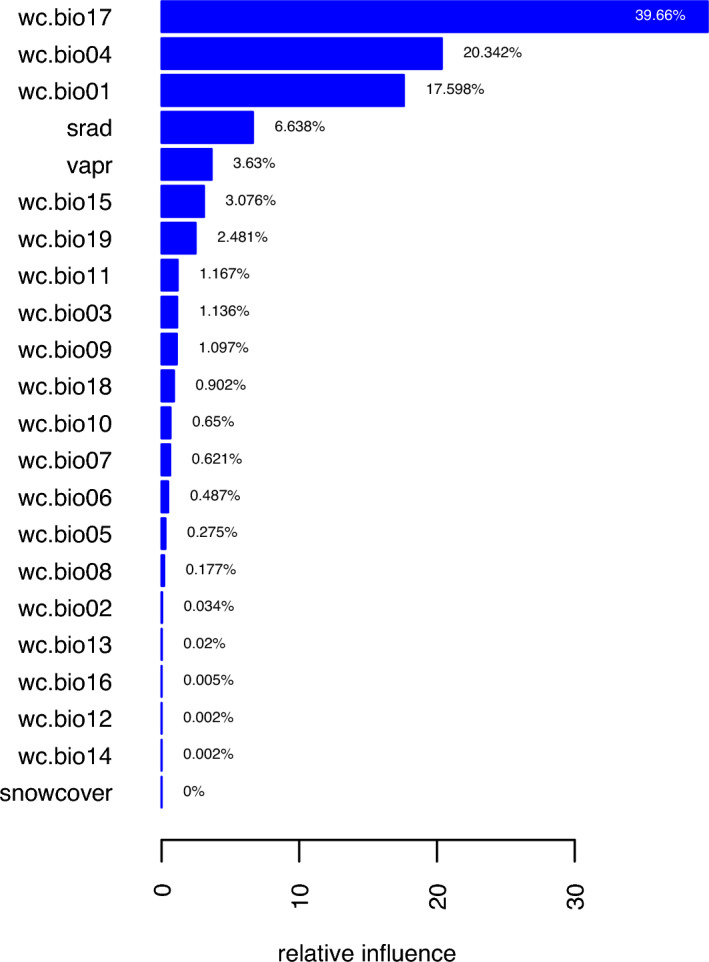
The relative influence of the predictors in Himalayan musk deer distribution; predictors are listed on y-axis. We constructed our model with 22 potential predictors (see Table 2 for the complete list and full name of the abbreviations).

We found that various competing models we trained were slightly but noticeably different both in their predicted surface of probability as well as their accuracies which varied with background sampling schemes (AUC = 0.97 for 0.1 bias, and 0.99 for biases of 0.5, 0.75 and no bias). Based on AUC scores and our knowledge of Himalayan musk deer distribution and ecology^21,28,31^, we used a background sampling bias of 0.5 for all reported results. In descending order, the most important predictors (with > 5% relative influence) of Himalayan musk deer range identified by this model were: precipitation of driest quarter, temperature seasonality, annual mean temperature, and solar radiation (Fig. 2).

### Predicted distribution under current climatic conditions

**Figure 3:**
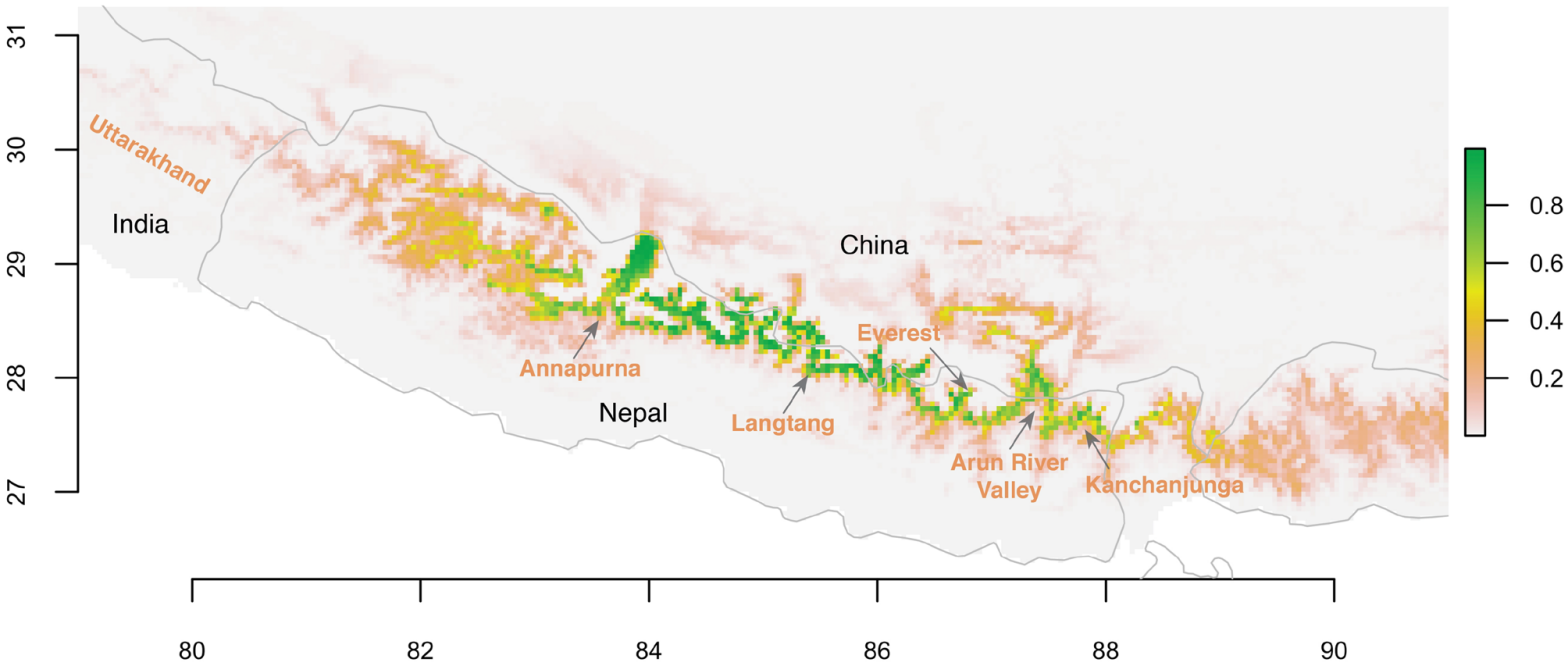
Habitat suitability estimated by species distribution models built with Maximum Entropy (MaxEnt) models. Habitat suitability is displayed as a continuous quantity between zero and one. The map was plotted using R 3.4.3 (R Foundation for Statistical Computing, Vienna, Austria, http://www.r-project.org/).

Our model predicted that Himalayan musk deer inhabits a continuous belt of the high Himalaya from Annapurna in central Nepal to Kanchenjunga Conservation Area (hereafter Kanchenjunga) in eastern Nepal, with some isolated patches further east of the Nepal–India border (Fig. 3). The model also predicted suitable habitats in Tibet along the Nepal-China border.

We categorized the continuous probability surface produced by the model into four categories: unsuitable (probability <0.2), marginally suitable (probability 0.2–0.5), suitable (probability 0.5–0.7), and highly suitable (probability > 0.7)^21,44–46^. Highly suitable and moderately suitable habitats are distributed continuously between Annapurna and Kanchenjunga including some regions of Tibet (Fig. 3, Supplementary Fig. S1). A substantial amount of highly suitable habitat was also predicted in Tibet, north of Arun River in eastern Nepal. A few patches of suitable habitat occur to the west of Annapurna which supports the closely related Kashmir musk deer (*M. cupreus*)^21^.

### Future distribution of Himalayan musk deer

**Figure 4:**
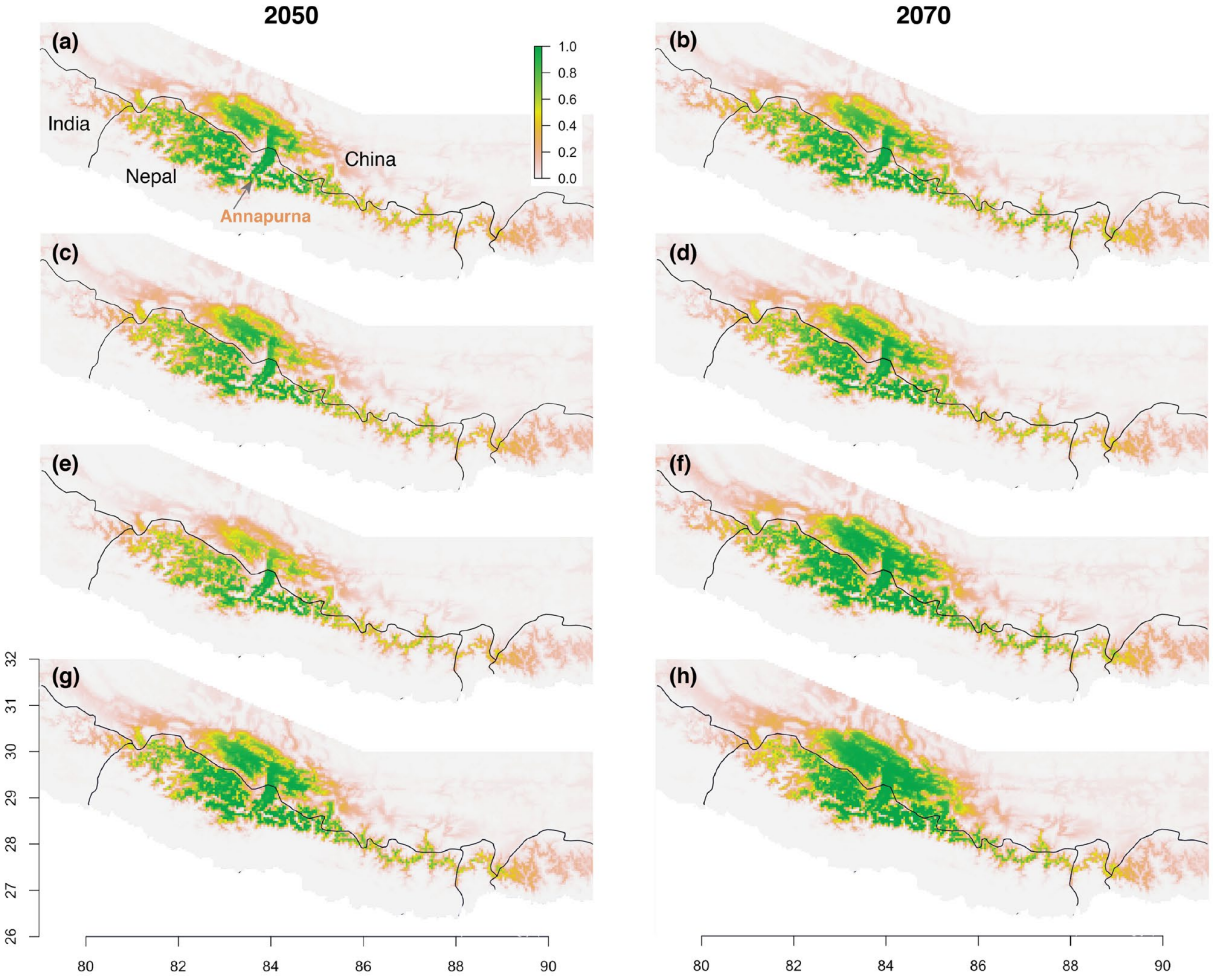
Estimated habitat suitability in 2050 and 2070 under various climate change scenarios. The prediction was made for the entire Himalaya (see Fig. 2), but we only show the part that includes all sites with suitable habitat. (**a**) RCP 2.6 climate scenario in 2050s. (**b**) RCP 2.6 climate scenario in 2070s. (**c**) RCP 4.5 in 2050s. (**d**) RCP 4.5 in 2070s. (**e**) RCP 6.0 in 2050s. (**f**) RCP 6.0 in 2070s. (**g**) RCP 8.5 in 2050s. (**h**) RCP 8.5 in 2070s. All maps were plotted using R 3.4.3 (R Foundation for Statistical Computing, Vienna, Austria, http://www.r-project.org/).

**Figure 5:**
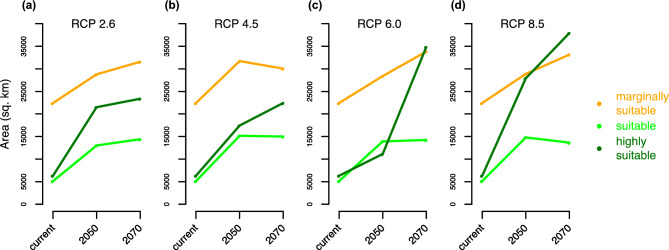
Change in area of suitable and marginally suitable habitats in future (probability: 0.2–0.5 for “marginally suitable”, 0.5–0.7 for “suitable”, and >0.7 for “highly suitable” habitats). The total area of all three types of suitable habitats was computed for all climate scenarios: RCP 2.6 (**a**), RCP 4.5 (**b**), RCP 6.0 (**c**) and RCP 8.5 (**d**). The area each grid cell was calculated separately from a raster in the Geographic Coordinate System accounting for Earth’s curvature, rather than in a flat Cartesian coordinate system. Marginally suitable habitats are not considered in the remainder of the analysis.

A vast majority (85–89%) of currently suitable habitat (probability > 0.5) is expected to remain suitable in the future under all climate scenarios (Fig. 4). However, the extent of suitable habitat to the east of Langtang was projected to decrease under all climate change scenarios. The suitable habitat of Himalayan musk deer was also predicted to expand west and north of the current extent by 2050 and 2070 under all climate change scenarios (Fig. 4). Most of Nepal’s mountainous regions west of Annapurna were predicted to harbor suitable habitat for Himalayan musk deer in the future under all four scenarios of climate change. With the expansion of suitable habitat to the west and north of Annapurna (Fig. 4), the total area of all suitable habitats (both “suitable” and “highly suitable”) increased in all future climate scenarios. In each scenario, the extent of highly suitable habitat (probability > 0.7) increased more than suitable habitat (0.5 < probability < 0.7). The area of suitable habitat was greatest in 2050, beyond which it remained largely constant, whereas the area of highly suitable habitat kept increasing after 2050 until 2070 (Fig. 5, Supplementary Fig. S2).

Overall, the predicted area of highly suitable habitat was greater than moderately suitable (Fig. 5), meaning the majority of predicted future suitable habitat (probability > 0.5) would be highly suitable. This proportion was predicted to be even greater west of Langtang. The predicted future increase in highly suitable habitat was more dramatic under scenarios RCP 6.0 and 8.5 than RCP 2.6 and RCP 4.5. In all future climate change scenarios, highly suitable habitat was predicted to dramatically increase west of Langtang, north and east of Annapurna as well as adjacent areas in Tibet (Supplementary Fig. S2).

### Protected areas and climate refugia

**Figure 6:**
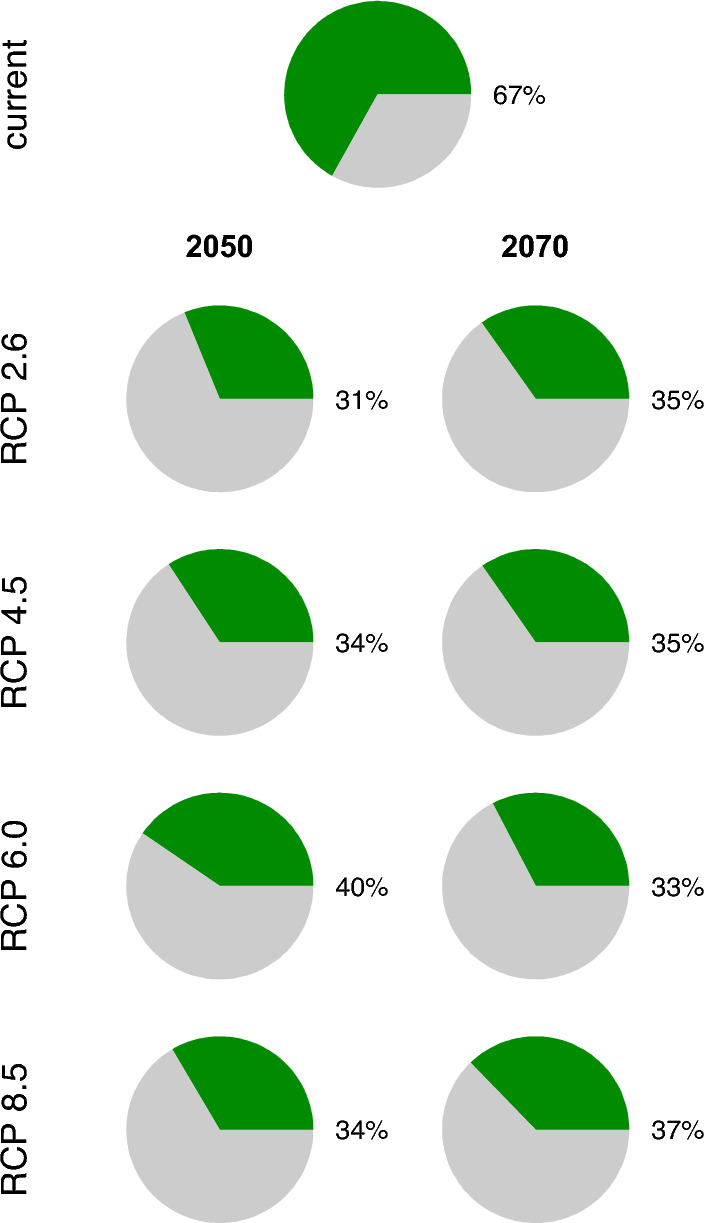
Fraction of the projected species habitat that is inside currently designated protected areas (shown in green shade). The species habitat is the totality of grid cells that have the probability of at least 0.5; this includes “suitable” and “highly suitable” habitats. This fraction was computed as the area of the habitat inside a protected areas divided by the total habitat area.

Two-thirds of the predicted suitable Musk deer habitat under current climate conditions are protected under currently designated network of protected areas (Fig. 6). As new suitable habitats develop to the west and north of the species current range, a smaller fraction of the future suitable habitat (31–40%) will be protected by the current networks of protected areas.

**Figure 7:**
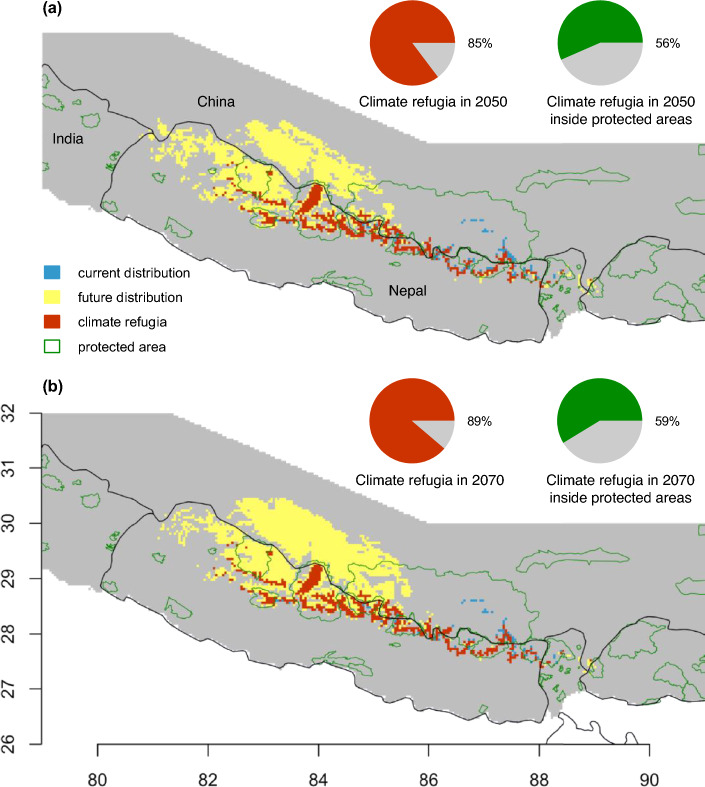
Climate refugia and protected areas. Climate refugia are assumed to occur where the current species habitat (“suitable” and “highly suitable” habitats aggregated, or all sites with probability of at least 0.5) overlaps with the predicted future habitat. Spatial distributions of climate refugia for 2050s (**a**) and for 2070s (**b**) under RCP 8.5 are shown. Next to each of these maps are two pie charts that display what fraction of the species current habitat remains as a potential climate refugia (red pie) and the fraction of that climate refugia that is located inside protected areas (green pie). All maps were plotted using R 3.4.3 (R Foundation for Statistical Computing, Vienna, Austria, http://www.r-project.org/).

Wherever current and future habitats overlap, such areas provide higher conservation value and can act as climate refugia. We found that 85% and 89% of current suitable habitat would serve as climate refugia in 2050 and 2070, respectively (Fig. 7). Over half of the climatic refugia (56% in 2050 and 59% in 2070) would be located inside current protected areas.

## Discussion

### Predictors of species distribution

The best-fitting species distribution models show that the distribution of Himalayan musk deer is determined primarily by three factors: precipitation of the driest quarter, temperature seasonality and annual mean temperature (Fig. 2). These influences on the distribution of musk deer should be considered in the context of their effects on vegetation because the habitat suitability of musk deer strongly depends on vegetation^27,28,39,42,47^. In the Himalaya, tree growth is largely influenced by moisture availability from melting snow in the pre-monsoon season (i.e. April to May), which is the driest period of the year^48–52^. Availability of water is important for vegetation, and different forms of precipitation such as rain and snowfall are the main drivers of vegetation structure, dynamics and processes worldwide^53–55^. However, determining the cause of species distribution is beyond the scope of this study. A correlative species distribution model is a predictive model that utilizes several variables for a robust prediction of species distribution. Due to varying degrees of correlation among the predictor variables, a predictor deemed highly important by the model incorporates some of the predictive ability of correlated variables. In our study, it is reasonable to argue that although precipitation of the driest quarter was the most important driver of animal distribution, that likely resulted from the possibility that the precipitation of the driest quarter along with other important or correlated variables influence the type, structure, density and other attributes of vegetation on which the animals directly depend.

Our finding that precipitation was the most important predictor of Himalayan musk deer distribution is in contrast to prior studies, viz. Khadka et al.^25^, Khadka and James^26^, Lamsal et al.^22^. In these studies, Musk deer distribution was best explained by temperature, although the authors used the same WorldClim (http://www.worldclim.com) climatic predictors we used. For example, Khadka et al.^25^ reported a 71% relative influence of annual mean temperature whereas Lamsal et al.^22^ found that annual mean temperature, elevation (which is fairly strongly correlated to temperature^56^) and isothermality were the top three predictors with relative influence of 47%, 16% and 14%, respectively. The differences in important predictors between our analyses and these previous works highlight the influence and importance of accurate and systematic occurrence data to building reliable species distribution models. These prior studies used taxonomically misidentified occurrence data because the results of recent genetic analysis were not available.

Because these prior studies ended up with a model that is enormously influenced by temperature related variables, they identified vast stretches of the high Himalaya including barren tall mountains and deserts of Tibet as current suitable habitat for Himalayan musk deer. The ecology and behavior of the animal requires forest and shrubs with reasonable density and canopy in its habitats^27,28,39,42,47^. The animals are shy in nature and prefer to hide in vegetation^28^, which does not exist higher than 6000 MASL or in the deserts of Tibet. On the contrary, our predicted suitability surface is highly heterogeneous (something expected for a highly heterogeneous and rugged surface of the mountains), avoids predicting suitable habitats in ice-capped mountains and the deserts of Tibet, and has a very close match to expert knowledge about distribution, ecology, and behavior of the species. Specifically, our finding of moisture being the most important predictor of the distribution aligns to the idea of how indirect, but strong, influence can be exerted by precipitation on animal distribution through its influence on structure of vegetation^53–55^.

### Current distribution of Himalayan musk deer

This analysis contributes important information to the conservation of Himalayan musk deer by identifying a much reduced range than previously considered. Our analysis indicates that the current distribution of Himalayan musk deer is limited to Nepal and Tibet. This is much smaller than the range of Himalayan musk deer described by the International Union for Conservation of Nature (IUCN), which includes parts of Bhutan, Nepal, China and northern India^9,12^. The IUCN species range published in 2015 shows Himalayan musk deer distributed from Bhutan on the east to Kashmir of India on the west^11^ (https://www.iucnredlist.org/species/13901/61977764). The IUCN also unrealistically indicates that the eastern extent of Kashmir musk deer range lies in Kashmir^11,12^ (https://www.iucnredlist.org/species/136750/61979453). In contrast, our findings align with recent genetic analyses of musk deer populations^9,19^, which indicate that the western extent of Himalayan musk deer range is in  the Annapurna region of Nepal, while the eastern extent of Kashmir musk deer range is situated to the west of the Annapurna region. The current distribution of Himalayan musk deer predicted by our analysis is located within the range of genetically confirmed populations of the species^9,57^. Singh et al. genetically confirmed  the Annapurna region in Nepal as the western limit of Himalayan musk deer^9^, and Guo et al. genetically confirmed that the population of musk deer to the north of Kanchenjunga in eastern Nepal (in Tibet) is of Himalayan musk deer^57^. Our analysis indicates that Kanchenjunga is the eastern limit of Himalayan musk deer range. Another study conducted in Bhutan reported the presence of both Alpine musk deer and Himalayan musk deer in Jigme Dorji National Park, Bhutan^58^. Unfortunately, this study also relied on pelage color to identify species of musk deer, which is an unreliable criteria for distinguishing species due to substantial intraspecific variation within population^9,18^ and individual variation among seasons^9^. Our model does not predict any suitable habitats in Bhutan. Collectively, previous range estimates, including those used by the IUCN, overestimated the extent of Himalayan musk deer.

Since 1994, the IUCN has been assigning conservation status to plants and animals, categorizing species as either extinct, critically endangered, endangered, vulnerable, and least concern^59^. The range size of a species is a major criterion in determining its conservation status^60^. Therefore, it is concerning that previous studies have grossly overestimated the Himalayan musk deer range^11,12^, including predicting suitable habitats above the Himalaya snow line and in dry Tibet—areas lacking the required vegetation for the Himalayan musk deer^25,26^. Overestimating the range of an endangered species can jeopardize conservation efforts by giving an incorrect impression that a species is more secured than it is. Our results, with an ecologically realistic and considerably smaller range compared to prior studies, can be used to update the current status of Himalayan musk deer, information that is crucial for prioritizing conservation resources and efforts.

### Future distribution of Himalayan musk deer

In 2050s and 2070s, suitable habitat is projected to extend to the west and north of the current western boundary of Himalayan musk deer range, expanding its size and extent. At the same time, the current range in the eastern half of Nepal Himalaya is likely to retain most of the currently suitable habitat. As we predicted a dramatic increase of habitat in new sites west of Langtang region and west and north of Annapurna region in the future, it is important to emphasize that the diverse topography of Himalaya creates complex climate systems which determine the vegetation structure as well as the habitat suitability for Himalayan musk deer.

Interestingly, new suitable habitats predicted to appear in Tibet in future climate scenarios are located in river valleys that crosscut the Himalaya. The Kaligandaki River valley located in the Annapurna region starts at the Nepal-China border and crosscuts Annapurna and Dhaulagiri Himalaya. The Trishuli River—called Gyirong River in Tibet—cuts through the Himalaya in the Langtang region and the Arun River bisects Himalaya east of Mount Everest. Typically, moisture-laden air moving north from the Bay of Bengal loses its moisture when crossing the Himalaya due to the cold temperatures at high altitudes, rendering Tibet a very dry land^61^. However, these river valleys serve as narrow lower-elevation passages crossing the Himalaya, allowing moist air to reach the other side of the mountain range. As a result, these regions currently harbor different types of temperate and alpine forests^62,63^. Thus, it is not surprising that some of the future suitable sites of Himalayan musk deer are likely to emerge in Tibet, adjacent to these river valleys.

A recent study^21^ has shown distribution of Kashmir musk deer (*M. cupreus*) west of Annapurna region. Himalayan musk deer and Kashmir musk deer are sister species of family *Moschidae*. At present, their ranges do not overlap but are separated at Annapurna-Kaligandaki region. Our study shows that the future westward expansion of range of Himalayan musk deer will bring it to the predicted future species range of Kashmir musk deer^21^. As the current mutually exclusive species ranges of two species will give way to a degree of overlap in distribution in future, it is hard to predict the dynamics of these two closely related species when they coexist in future.

### Protected areas and climate refugia

For Himalayan musk deer, a large portion (67%) of currently suitable habitat (probability > 0.5) is located within protected areas. Compared to this, the protection for Kashmir musk deer is dismally low, with just 17% of suitable habitat being protected^21^. The reason for this discrepancy is that most of the Himalaya in eastern Nepal as well as in Tibet are protected whereas that is the not case with western Nepal. A recent study^21^ has demonstrated that the range of Kashmir musk deer (*M. cupreus*) extends to the west of the Annapurna region. Himalayan musk deer and Kashmir musk deer are sister species of family *Moschidae*. At present, their ranges do not overlap; instead they are separated in the Annapurna-Kaligandaki region. Our study shows that the future westward expansion of Himalayan musk deer range will create overlap with the predicted future range of Kashmir musk deer^21^. The future westward expansion of Himalayan musk deer distribution will result in most of the future suitable habitat falling out of protected areas. Specifically, only 31 to 40% of all the future suitable habitat predicted under various climate change scenarios will be protected under presently designed protected areas (Fig. 7). By incorporating future suitable habitat for Himalayan musk deer in the design and prioritization of protected areas, managers can provide conservation benefits for both species.

The vast majority of current Himalayan musk deer habitat will serve as climate refugia in the future (85 to 89% in different climate change scenarios). A majority of climate refugia (56 to 59%) will be protected inside currently designated network of protected areas. Climate change refugia are generally expected to represent only a small fraction of a species' current distribution^64^, owing to the rapid rate of climate change and land use change globally. However, in our study, a significant portion of the current distribution of Himalayan musk deer is predicted to continue supporting the species' distribution in the near future. Additionally, our study also indicates the emergence of new suitable sites to the west of the species' current range. As a result, this species may be more resilient to future climate change compared to other imperiled species. We propose two plausible explanations for this phenomenon. First, the most important predictor of Himalayan musk deer distribution—precipitation of the driest quarter—does not necessarily follow the simpler elevational trends of temperature. Instead, it remains favorable in the current species range but becomes even more favorable in the western half of the Nepal Himalaya. Second, the next two most important variables are related to temperature. In mountain systems, climate velocities are lower, causing the climatic envelope of a species to shift by short distances^65^. Although a simple linear relationship between a predictor variable and habitat suitability should not be expected under the MaxEnt modeling framework, the identification of the most crucial predictors aids in understanding of ecological basis of species distribution.

## Conclusion

Identification of various species of musk deer in the Himalaya has been plagued by the usage of inaccurate methods for a long time. However, recent genetic studies^9,19,20,23,24,66^ have successfully confirmed the populations of  various species of musk deer across the Himalaya. We took advantage of these recent genetic findings and collected high-quality occurrence records of latrine sites from systematic surveys (rather than relying on opportunistic sightings), and constructed species distribution models. Our stud reveals a future westward and northward expansion of species distribution that will lead to the overlapping of  currently spatially separated Himalayan musk deer and Kashmir musk deer ranges. The interaction dynamics between these two closely related species will influence their coexistence in the future. This can potentially bring challenges for conservation because Kashmir musk deer will have much smaller climate refugia protected under protected areas than Himalayan musk deer. Our findings highlight the importance of reviewing the expert opinion-driven IUCN map, which indicated a larger range for Himalayan musk deer than our predicted substantially smaller range.

The optimistic conservation scenario for Himalayan musk deer (substantial future range expansion and vast majority of current range serving as climate refugia) should be viewed against the backdrop of the strong force of poaching that has rapidly dwindled the musk deer population in recent decades. The new suitable habitats that are predicted to appear in the western Nepal Himalaya will ensure conservation if they are protected. Himalaya is inhabited by four species of musk deer. Given the way the species were traditionally identified with unreliable indicator of pelage color, it is likely that the taxonomic misidentification is a problem for other species as well. Genetic analysis should be employed to address these uncertainties and provide reliable and quantitative evidence for reviewing the species range of various musk deer species. We call for a concerted effort of national and international agencies for a review of species range of various musk deer species based on reliable and quantitative evidences.

## Material and methods

### Study area and occurrence records

**Figure 8:**
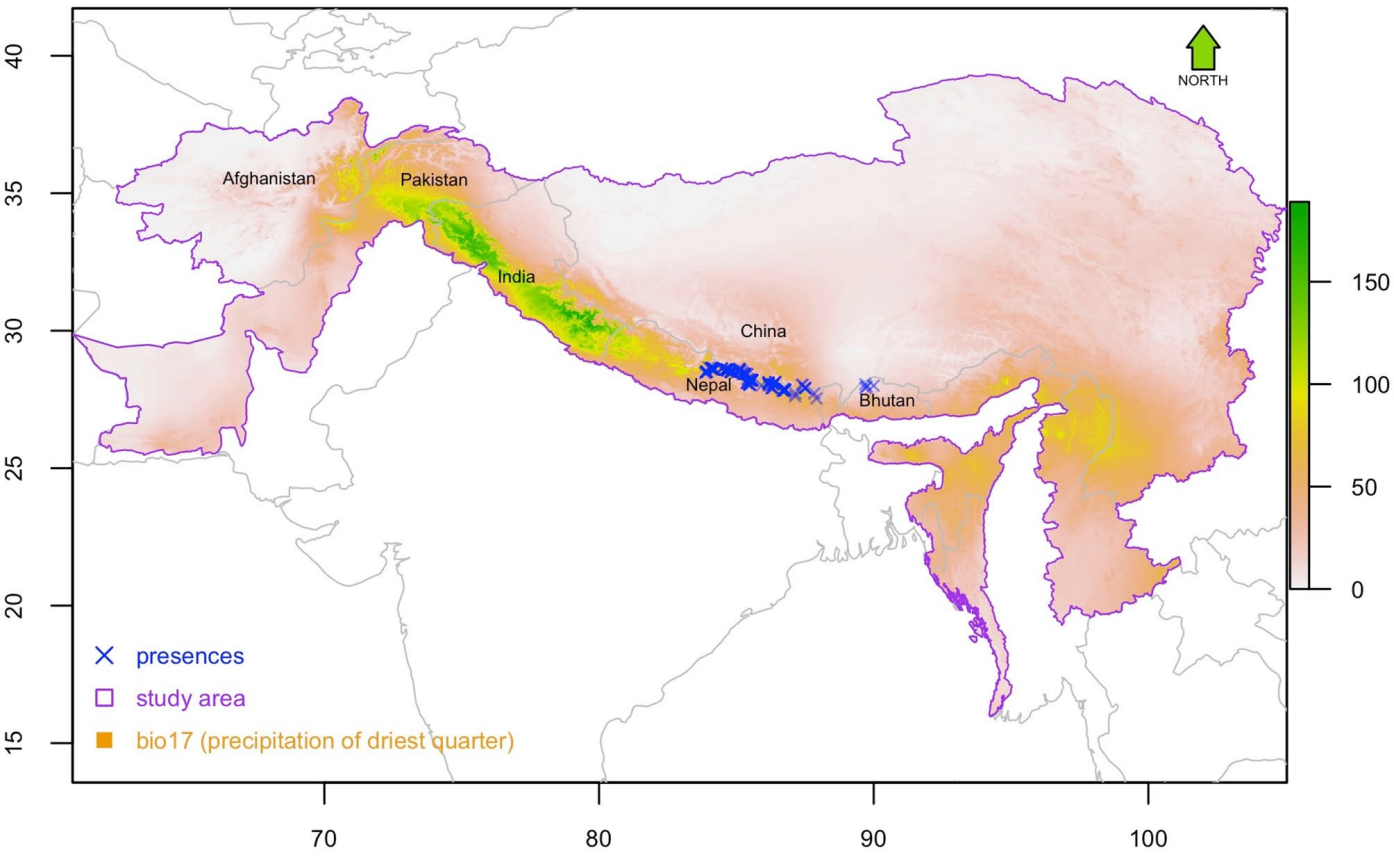
Occurrences ( a total of 294) of Himalayan musk deer against the backdrop of precipitation of the driest quarter, which was identified by this study as the most important predictor of Himalayan musk deer distribution, in the study area (purple polygon) where the model training and prediction was performed. The map was plotted using R 3.4.3 (R Foundation for Statistical Computing, Vienna, Austria, http://www.r-project.org/).

We performed species distribution modeling of Himalayan musk deer across Himalaya by using occurrences records collected from high quality habitats with systematic sampling. The Himalaya includes four (completely or partially) of the 36 global biodiversity hotspots: Himalaya, Indo Burma, Mountains of Southwest China, and Mountains of Central Asia^67–69^. The Himalaya can be approximately divided into wetter eastern and drier western forests by Nepal’s Kaligandaki River Valley^70,71^ (see the background surface of Fig. 8 for precipitation of driest quarter). Recent genetic analyses of the multiple populations of musk deer from southern and northern parts of Himalaya confirmed that Himalayan musk deer exist in the eastern half of Nepal as well as some adjacent parts of Tibet, an autonomous region of China (hereafter Tibet)^9,19,21,66^. The current western boundary of Himalayan musk deer range is Annapurna Himalaya (hereafter Annapurna). Further west of this boundary lies the eastern boundary of a closely related species Kashmir musk deer (*M. cupreus*) at Kaligandaki valley. We conducted multiple systematic field surveys to collect geographical location of latrine sites within the species range delineated based on the genetic evidences of multiple studies^9,19,57^.

Latrine sites are not just for defecation. They have multiple purposes including communication among conspecifics and territory marking. Therefore, latrine sites are always located in high quality habitats—areas with suitable environmental conditions and habitat structure. Apart from being high quality habitat, latrine sites are also confirmed and irrefutable evidence of species occurrence. An animal can be spotted in unsuitable sites while roaming around in search of isolated patches of suitable habitats. Inclusion of such unsuitable habitats as species occurrence sites is not helpful in building an efficient model. Therefore, our main occurrence data includes latrine sites which represent confirmed locations of suitable habitats.

We collected 294 occurrence records from nine protected areas, seven of them in Nepal, one each in China and Bhutan (Fig. 8, Table 1). We used the location of latrine sites for the vast majority of our occurrence data (273, 93% of the total), although we include some records (21, 7%) of survey-based and expert guided sightings of animals. The majority (98.7%) of the occurrences (290 out of 294) were collected in the field for this study (primary data) and 90% (261 points out of 294) were collected using systematic sampling. To represent diverse habitats in our data, we surveyed various river valleys located within each protected area. The forests we visited fell into two broad categories: broadleaf and conifer forests. The dominant species of broadleaf forests included rhododendron (*Rhododendron* spp.) and oak (*Quercus* spp.) at the lower elevations within the species range (~ 2500 to 3200 m) and Himalayan birch (*Betula utilis*) at higher elevations (~ 3500 to 4200 m). The conifer forests were comprised of blue pine (*Pinus wallichiana*; ~ 2000 to 3800 m), west Himalayan fir (*Abies spectabilis*; ~ 2700 to 4000 m) and east Himalayan fir (*Abies pindrow*; ~ 2700 to 4000 m) as the dominant species.

**Table 1:** Occurrence records of Himalayan musk deer utilized in the current study to construct species distribution models*.*

Country	Protected area	Primary collection of occurrences		Secondary collection of occurrences	Method of data collection
		Latrine sites	Sighting of animal	Retrieved from literature	Systematic survey
Nepal	Annapurna Conservation Area (ACA)	53	4		Systematic survey
	Manaslu Conservation Area (MCA)	63	2		Systematic survey
	Langtang National Park (LNP)	93	7		Systematic survey
	Gaurishankhar Conservation Area (GCA)	13	1		Systematic survey
	Sagarmatha National Park (SNP)	23	2		Systematic survey
	Makalu Barun National Park (MBNP)	4			Opportunities sighting
	Kanchenjunga Conservation Area (KCA)	2			Opportunistic sighting
China	Qomolagma National Nature Preserve (QNNP)	22	1		Visiting various patches based on expert knowledge
Bhutan	Jigme Dorji National Park (JDNP)			4 (animal location)	Secondary source
Total		273 Systematic survey = 245 Opportunistic sightings = 6 Expert guided = 22	17 Systematic survey = 16 Expert guided = 1	4 Secondary source = 4	

In five protected areas, we employed systematic surveys between 2500 and 4300 MASL (Table 1). In each protected area, we selected six patches of forest ranging between 5 and 11 km^2^ as the study sites; inside these study sites, which were separated by at least 1 km, we laid multiple transects. A study site included various forest types, i.e., Rhododendron forest, oak forest, pure blue pine forest, Himalayan birch forest, mixed forest of blue pine and fir, and mixed forest of fir and Himalayan birch. In each forest type, at least 3 transects of 0.5 km length each were laid along the elevational gradient, keeping an elevational gap of 200 m between two transects. As we walked along the trail, we inspected the forest on both sides and recorded location of latrines when observed. In two protected areas, viz. Makalu Barun National Park (MBNP) and Kanchenjunga Conservation Area (KCA), occurrence records (7% of total occurrence data) were based only on opportunistic sightings of latrine sites by game scouts and rangers while patrolling musk deer habitat in those protected area. In Qomolagma National Nature Preserve (QNNP), various types of forest at different elevation were visited to find fresh latrine sites. A total of 22 latrine sites and one animal location were identified in those locations through an expert-guided search. In the case of Jigme Dorji National Park (JDNP), presence locations of animal were extracted by scanning and georeferencing previously published range maps^58^. The populations in ACA, GCA and QNNP are genetically confirmed to be of Himalayan musk deer^9,19,66^. The other protected areas of Nepal fall in between these protected areas.

Our sampling happened to be exclusively inside protected areas because the vast majority of the high Himalaya within the Himalayan musk deer range is protected. The surveyed and sampled protected areas in Nepal, China and Bhutan cover almost the entire east–west range of the species with the following exceptions: geographic space between MCA and LNP and the between MBNP and KCA, both regions being in Nepal, were not sampled; see the map of protected areas on Nepal Government’s Department of National Park and Wildlife Conservation website (http://www.dnpwc.gov.np). These areas do not fall under protected areas because the vast majority of these gaps between protected areas consist of permanent snow and ice. Therefore, virtually all of the species range in Nepal was sampled for occurrences. Further east of Nepal, the species range extends up to Bhutan. We have obtained occurrences from Bhutan’s protected area. This leaves a narrow space of India between Nepal and Bhutan, which is 45 to 86 km wide, unsampled. This space falls in the continuum of geography, environment, and habitat types where both sides of the space are sampled. Hence it is reasonable to argue that this narrow unsampled space is represented in the multivariate environmental space of sampled locations. Nepal, where vast majority of the species range lies, contributes 267 (91%) of the occurrence records in our study (Table 1). These occurrences were distributed in most places of the high Himalaya of eastern half of Nepal. Therefore, the sampling strategy covered a representative sample of habitat types and geographic characteristics present within the entire species range.

### Potential predictors of species distribution

We obtained 19 bioclimatic covariates at 2.5 arc-minutes spatial resolution from WorldClim (Version 2.0) (http://www.worldclim.com) (Table 2). These standard covariates of species distribution include annual trends, seasonality, and variability in temperature and precipitation^72^. This set of variables was obtained for current climatic conditions as well as for four climate change scenarios (RCP2.6, RCP4.5, RCP6, and RCP8.5) for the year 2050s and 2070s as described in the Fifth Assessment by the International Panel on Climate Change^73^. Future values of the bioclimatic covariates at the same spatial resolution (2.5 arc-minute) projected with BCC-CSM1-1 model were obtained from Worldclim. BCC-CSM1-1 is a Global Circulation Model developed by Beijing Climate Center China based on Community Climate System Model (CCSM) to understand climate predictions and to assess impact at various time scale such as month, season, year, particularly over Asia^74,75^. We chose BCC-CSM1-1 after reviewing several prior published studies on GCM. Of all the models, we found BCC-CSM1-1, CCSM4, HadGEM2-CC and MIROC-5 more frequently used in SDM for future projection of habitat suitability^76,77^. In our case, BCC-CSM1-1 was selected because it is based on CCSM and is widely used for projecting future species distribution^75,78^.

**Table 2:** The potential predictors of Himalayan musk deer distribution provided as inputs to the species distribution models.

S. n	Variable abbreviation	Variable name
1	bio01	Annual mean temperature
2	bio02	Mean diurnal range
3	bio03	Isothermally
4	bio04	Temperature seasonality
5	bio05	Maximum temperature of warmest month
6	bio06	Minimum temperature of coldest month
7	bio07	Temperature annual range
8	bio08	Mean temperature of wettest quarter
9	bio09	Mean temperature of driest quarter
10	bio10	Mean temperature of warmest quarter
11	bio11	Mean temperature of coldest quarter
12	bio12	Annual precipitation
13	bio13	Precipitation of wettest month
14	bio14	Precipitation of driest month
15	bio15	Precipitation seasonality
16	bio16	Precipitation of wettest quarter
17	bio17	Precipitation of driest quarter
18	bio18	Precipitation of warmest quarter
19	bio19	Precipitation of coldest quarter
20	srad	Solar radiation
21	vapr	Water vapor pressure
22	snowcover	Snow cover

All except “snow cover” layers were obtained from WorldClim (version 2.0). Snow cover was obtained from International Center for Integrated Mountain Development (ICIMOD).

RCP 2.6 assumes carbon dioxide emissions decline beginning in 2020 and reach zero by 2100^79^. In this scenario global temperature rise is below 2 °C by 2100. RCP 4.5 predicts global temperature to rise between 2 and 3 °C by 2100, with emissions peaking in 2040 and declining thereafter. RCP 6 Scenario assumes emissions peak around 2080 and then decline; RCP 8.5 scenario is the worst case climate change scenario where temperature is likely to increase up to 4.8 °C^79,80^. We projected the future distribution of Himalayan musk deer in all these four emission scenarios in 2050 and 2070.

We supplemented this standard set of potential covariates with solar radiation, vapor pressure and snow cover (Table 2). Our initial expectation was that these variables might have some predictive power for species distribution. However, these potential predictors showed very limited predictive power (6.6%, 3.6%, and 0.0019% relative influence, respectively). It is important to note that these variables are likely to change with climate, although accurately estimating the expected changes is challenging (we could not find any reliable raster product of these variables during analysis). Despite this fact, the trivial contribution of these variables to the model made us believe that they could be used as potential predictors for future climate projections.

We excluded “altitude” as a predictor because altitude does not vary over time and the distribution of a species is not directly determined by altitude but by environmental variables correlated with altitude. Given the fact that Himalayan musk deer inhabit certain types of forest, it is tempting to use land cover as a predictor of its distribution. While this approach can be useful for predicting current distribution, it is problematic for projecting future distributions unless a comparable product of landcover for future exists. Because land cover is one of the features of natural world that is changing rapidly and is expected to continue the trend, we excluded this variable as a predictor of species distribution.

Our posthoc analysis revealed that all the strong collinearity (absolute value of correlation > 0.7) exist only between predictors with at least one of them being trivially important (relative influence ≤ 5%). A lack of strong collinearity between important predictors is crucial for model transferability into novel environments as shift in collinearity between such pairs likely makes the model unreliable^81^. We observed a lack of collinearity between two important predictors.

### Species distribution model

We used MaxEnt^82,83^ to predict the distribution of Himalayan musk deer at 2.5 arc-minute spatial resolution based on presence-only data. MaxEnt is one of the most efficient and robust methods for species distribution modeling (SDM)^84^, and is also a widely used method for estimating the extent of species ranges when presence-only data are available^30^. Like any statistical model used to estimate species distributions, MaxEnt has several limitations^31,85,86^. These include biased sampling, spatial autocorrelation in observations, use of default regularization parameter without critical evaluation, inadequate sampling of occupied locations failing to represent the environmental niche properly, etc. However, tuning a MaxEnt model based on the expertise of the ecology of a species and statistics yields a realistic habitat suitability map because there are fewer issues than regression models such as generalized linear models, random forest, boosted regression trees, etc. For instance, the binary presence-background data of species distribution (as is the case with most SDM studies because presence-absence data is rare) characterizes species presence on the geographic background. A model that allows presences to represent a subset of the background fits conceptually with the actual process of species distribution in presence-background or presence-pseudo absence data. MaxEnt and inhomogeneous Poisson point process (IPP) models operate with such assumptions^82,83,87,88^, making them most suitable for modeling species distribution with presence-only data. An assumption of the MaxEnt model is that occurrences are collected either randomly or systematically^35^. The vast majority of occurrences (90%) we collected were systematically sampled.

One way to improve model performance is by adjusting the background pseudo-absence sampling scheme so that occupied grid cells contribute same number of background points as the occurrences whereas the unoccupied grid cells contribute a background point that is drawn at lower probability^32,35^. We performed three schemes of biased background drawing with probabilities of drawing an unoccupied cell set at 0.1, 0.5 and 0.75^20^. Additionally, we also performed a completely random drawing of background points. In total, 10,000 background points were drawn under each scheme from the entire study area (purple polygon, Fig. 8). To determine which of these four sampling schemes resulted in the best performing model, we examined the predicted surface and used AUC on the held-out test dataset of a five-fold cross-validation. We performed 50 iterations of the models and averaged the outputs of habitat suitability, AUC, and variable importance. Although AUC has often been criticized for being sensitive to geographic background and other aspects of the modeling, we supplied the same set of presence points and the spatial extent for drawing background points to all models that we compared. Therefore, the issues of AUC likely affect all models similarly, allowing a reasonable comparison of background sampling schemes based on AUC.

### Categorization of habitat suitability

Delineating species ranges or suitable and highly suitable habitats requires selection of reasonable thresholds of probability values to delimit categories. Several prior studies including Guo et al.^44^, Singh et al.^21^, Olivero et al.^46^ and Okurut et al.^45^, followed similar but not identical cutoff values to delineate various categories of habitats. Based on these prior studies, we categorized the continuous probability surface of the output SDM into four categories: unsuitable (probability 0–0.2), marginally suitable (probability 0.2–0.5), suitable (probability 0.5–0.7), and highly suitable (> 0.7).

### Climate refugia

Climate change refugia are “generally defined as areas relatively buffered from contemporary climate change”^89^. This inclusive definition of climate change refugia can include anything that enhances species survival through time. For the purpose of this study, we use a strict definition of climate change refugia which can be defined as locations with analogous climatic conditions retained in place^90^. For practical purpose, this definition extends to habitats that continue to remain suitable (probability of at least 0.5) through time. We estimate the climate refugia through 2050 and 2070 as the fraction of the current suitable sites that continue to support suitable habitats in the future.

### Analysis

All analyses and visualizations were done with R 3.4.3 (R Foundation for Statistical Computing, Vienna, Austria, http://www.r-project.org/)^91^. The main analysis of SDM was performed with the packages “dismo”. The following packages were used in other analyses and plotting: “raster”, “sp”, “maps”, “rgeos”, “plyr”, “matrixStats”, “scales”.

### Supplementary Information

Supplementary Figures.

## Data Availability

Musk deer is an endangered species and poaching for the musk is major cause of population decline of the deer. Our presence records are location of latrine sites and it was found that poachers aim to locate a currently used latrine site to track the deer. The latrine sites are used repeatedly by musk deer for many years. Because our data can be used by poachers to locate the animals, we cannot make our presence data publicly available. However, we can provide these data upon the formal request from the researchers to Dr. Paras Bikram Singh at ecoparas@gmail.com.
