# Supplementary material for: A brighter shade of future climate on Himalayan musk deer Moschus leucogaster
Source: Sci Rep. 2023 Aug 7;13:12771. doi: 10.1038/s41598-023-39481-z (PMC10406878; doi:10.1038/s41598-023-39481-z)
Supplement: Supplementary file 1 — Supplementary Figures. [file 41598_2023_39481_MOESM1_ESM.docx]

**A brighter shade of future climate on Himalayan musk deer *Moschus leucogaster***

Kumar P. Mainali ^1, 2^, Paras Bikram Singh ^3, 4*^, Michael Evans ^2, 5^, Arjun Adhikari ^6^, Yiming Hu ^3^, Huijian Hu ^3*^

*^1^National Socio-Environmental Synthesis Center, Annapolis, Maryland, USA.^2^Conservation Innovation Center, Chesapeake Conservancy, Annapolis, Maryland, USA.^3^Guangdong Key Laboratory of Animal Conservation and Resource Utilization, Guangdong Public Laboratory of Wild Animal Conservation and Utilization, Institute of Zoology, Guangdong Academy of Science, Guangzhou 510260, China.^4^Biodiversity Conservation Society Nepal, Bagdol, Lalitpur.^5^Environmental Science and Policy Dept., George Mason University, Fairfax, VA, USA.^6^Department of Natural Resource Ecology and Management, Oklahoma State University, Stillwater, OK, USA*

*Corresponding author, email: ecoparas@gmail.com, 13922339577@139.com

**List of the supplementary figures**

Supplementary Fig. S1. Categorical habitat suitability of Himalayan musk deer. The continuous surface of probability was divided into four categories of habitat suitability as below: unsuitable (0–0.2), marginally suitable (0.2–0.5), suitable (0.5–0.7), and highly suitable (0.7–1.0). The probability surface was generated by species distribution models built with Maximum Entropy (MaxEnt) Models. Map was plotted using R 3.4.3 (R Foundation for Statistical Computing, Vienna, Austria, <http://www.r-project.org/)>

Supplementary Figs. S2. The prediction was made for the entire Himalaya (see Figure 3) but we only show a part of it that includes all sites with any kind of suitable habitat. The parts of the Himalaya not shown have only unsuitable habitat. (a) RCP 2.6 climate scenario in 2050s, (b) RCP 2.6 climate scenario in 2070s, (c) RCP 4.5 in 2050s, (d) RCP 4.5 in 2070s, (e) RCP 6.0 in 2050s (f) RCP 6.0 in 2070s, (g) RCP 8.5 in 2050s and (h) RCP 8.5 in 2070s. All of Map were plotted using R 3.4.3 (R Foundation for Statistical Computing, Vienna, Austria, <http://www.r-project.org/)>


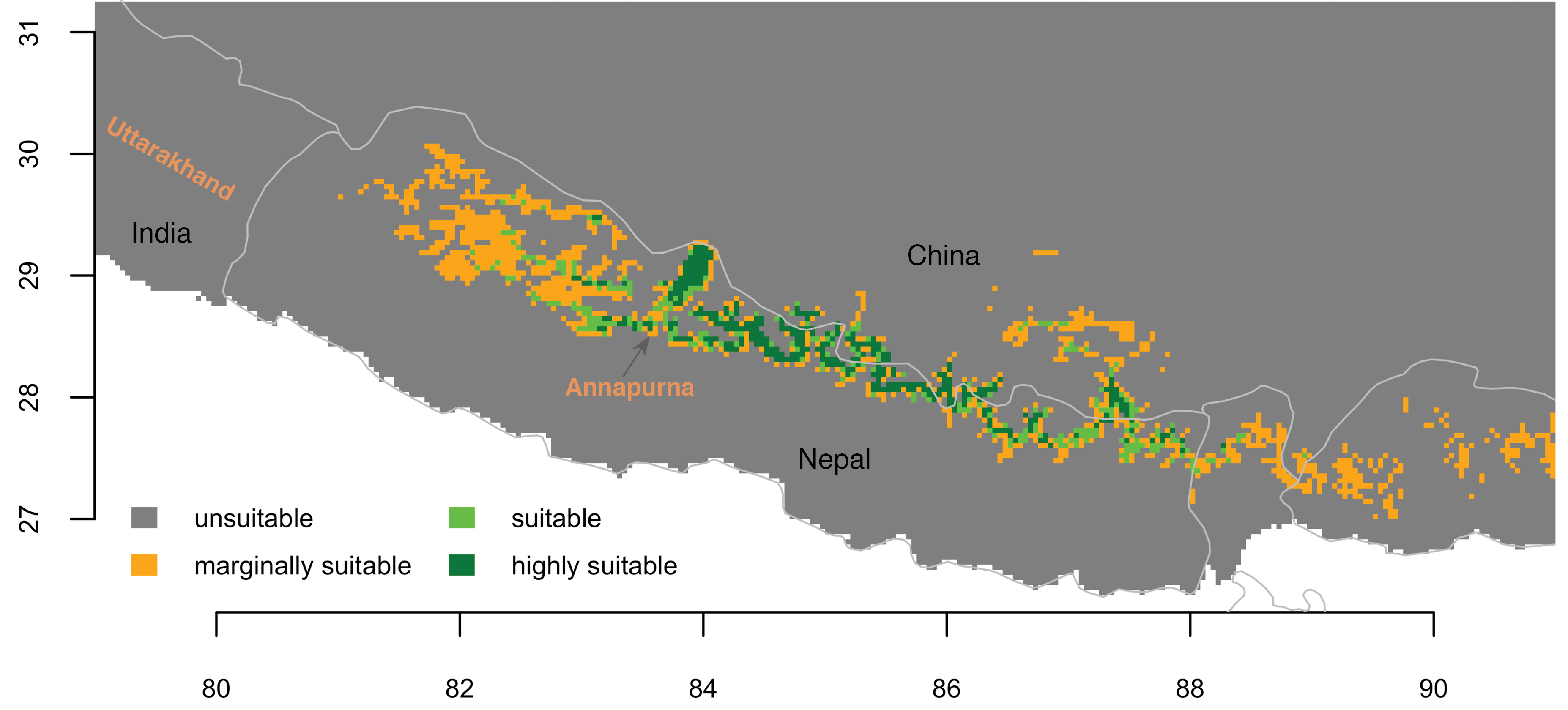


Supplementary Fig. S1. Categorical habitat suitability of Himalayan musk deer. The continuous surface of probability was divided into four categories of habitat suitability as below: unsuitable (0–0.2), marginally suitable (0.2–0.5), suitable (0.5–0.7), and highly suitable (0.7–1.0). The probability surface was generated by species distribution models built with Maximum Entropy (MaxEnt) Models. Map was plotted using R 3.4.3 (R Foundation for Statistical Computing, Vienna, Austria, <http://www.r-project.org/)>


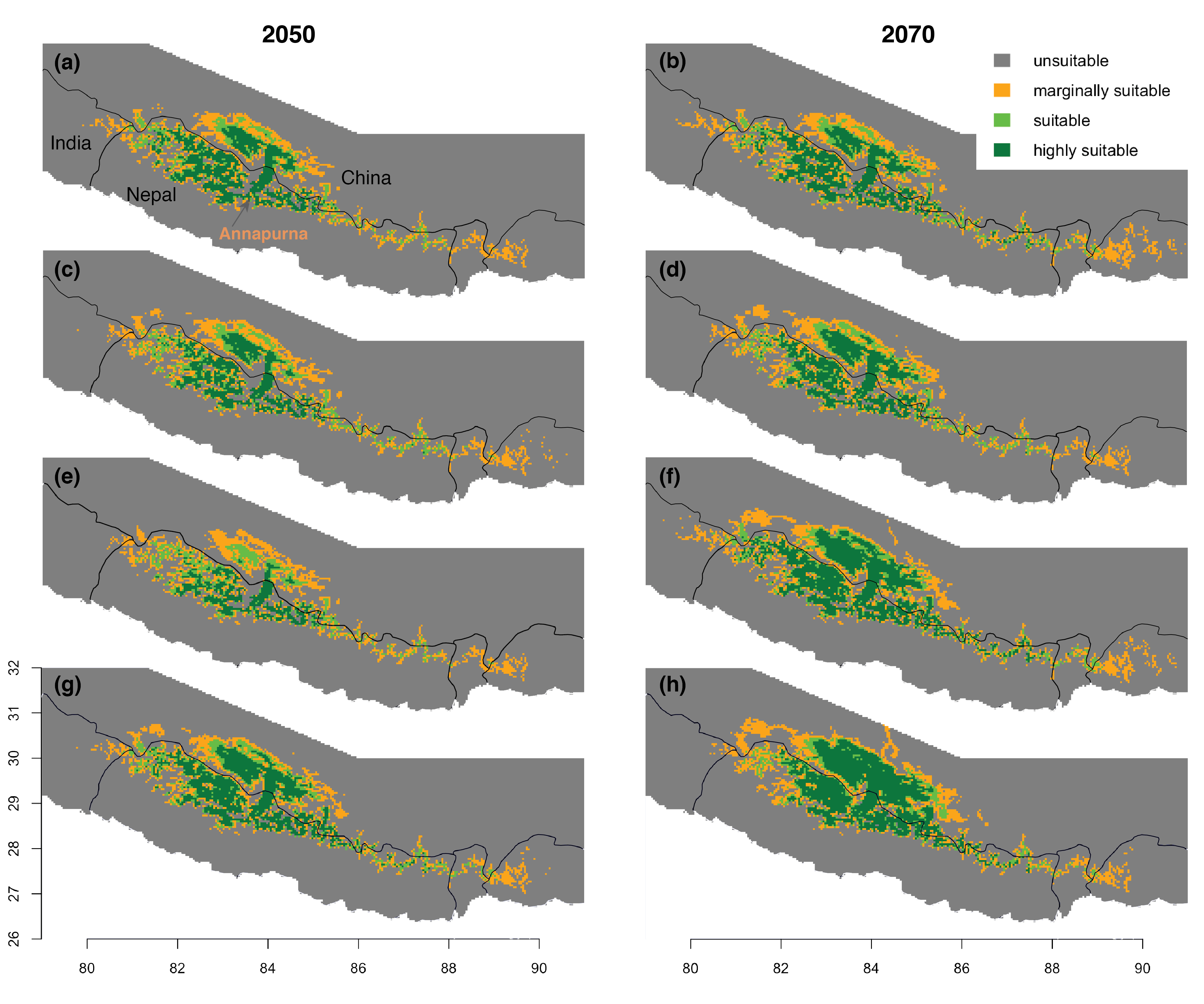


Supplementary Figs. S2. The prediction was made for the entire Himalaya (see Figure 3) but we only show a part of it that includes all sites with any kind of suitable habitat. The parts of the Himalaya not shown have only unsuitable habitat. (a) RCP 2.6 climate scenario in 2050s, (b) RCP 2.6 climate scenario in 2070s, (c) RCP 4.5 in 2050s, (d) RCP 4.5 in 2070s, (e) RCP 6.0 in 2050s (f) RCP 6.0 in 2070s, (g) RCP 8.5 in 2050s and (h) RCP 8.5 in 2070s. All of Map were plotted using R 3.4.3 (R Foundation for Statistical Computing, Vienna, Austria, <http://www.r-project.org/)>
